# Genome-wide DNA methylation analysis of human brain tissue from schizophrenia patients

**DOI:** 10.1038/tp.2013.111

**Published:** 2014-01-07

**Authors:** L F Wockner, E P Noble, B R Lawford, R McD Young, C P Morris, V L J Whitehall, J Voisey

**Affiliations:** 1 QIMR Berghofer Medical Research Institute, Brisbane, QLD, Australia; 2 Department of Psychiatry and Biobehavioral Sciences, University of California, Los Angeles, CA, USA; 3 Institute of Health and Biomedical Innovation, Queensland University of Technology, Brisbane, QLD, Australia; 4 Alcohol and Drug Service, Royal Brisbane and Women's Hospital, Brisbane, QLD, Australia

**Keywords:** brain, DNA methylation, epigenetics, schizophrenia

## Abstract

Recent studies suggest that genetic and environmental factors do not account for all the schizophrenia risk, and epigenetics also has a role in disease susceptibility. DNA methylation is a heritable epigenetic modification that can regulate gene expression. Genome-wide DNA methylation analysis was performed on post-mortem human brain tissue from 24 patients with schizophrenia and 24 unaffected controls. DNA methylation was assessed at over 485 000 CpG sites using the Illumina Infinium HumanMethylation450 Bead Chip. After adjusting for age and post-mortem interval, 4641 probes corresponding to 2929 unique genes were found to be differentially methylated. Of those genes, 1291 were located in a CpG island and 817 were in a promoter region. These include *NOS1*, *AKT1*, *DTNBP1*, *DNMT1*, *PPP3CC* and *SOX10*, which have previously been associated with schizophrenia. More than 100 of these genes overlap with a previous DNA methylation study of peripheral blood from schizophrenia patients in which 27 000 CpG sites were analysed. Unsupervised clustering analysis of the top 3000 most variable probes revealed two distinct groups with significantly more people with schizophrenia in cluster one compared with controls (*P*=1.74 × 10^−4^). The first cluster composed of 88% of patients with schizophrenia and only 12% controls, whereas the second cluster composed of 27% of patients with schizophrenia and 73% controls. These results strongly suggest that differential DNA methylation is important in schizophrenia etiology and add support for the use of DNA methylation profiles as a future prognostic indicator of schizophrenia.

## Introduction

Despite schizophrenia being a debilitating disorder affecting 1% of the population, there are no extant biomarkers to aid the clinician in identifying this disorder. Studies predict the genetic risk to be up to 80%,^1,2^ but despite strenuous research efforts the genes and polymorphisms found to be associated with schizophrenia account for very little of the genetic risk. Environmental risk such as urbancity,^3^ migrant status,^4^ childhood maltreatment,^5^ prenatal infections,^6^ cannabis use^7^ and maternal vitamin D deficiency^8^ also contribute to schizophrenia susceptibility. However, not all individuals exposed to environmental risk develop schizophrenia.^9^ This observation suggests that interaction between susceptibility genes and environmental factors may better account for schizophrenia. DNA methylation has been identified as a key mechanism for environmental regulation of gene expression.^10^ DNA methylation is an epigenetic modification that is essential for normal human development via regulation of gene function. DNA methylation results in the addition of a methyl group on the cytosine of CpG dinucleotides, which can then be inherited through cell division. These cytosine modifications can affect gene expression by altering the binding of transcription factors to promoter regions or changing mRNA processing.

DNA methylation studies of the brain and peripheral tissue have previously been reported for schizophrenia. However, to our knowledge, no study has published results from an Illumina Infinium HumanMethylation450 Beadchip in the brain tissue of patients with schizophrenia. Studies to date have typically been performed in peripheral tissues and have been limited to the analysis of CpG islands in the promoter regions. A recent DNA methylation study analysed 27 578 CpG sites in peripheral blood cells from 18 patients with schizophrenia and 15 normal controls.^11^ This study revealed 603 CpG sites (representing 589 genes) that had significantly different DNA methylation levels between schizophrenia and controls. Among these genes were *HTR1E*, *COMTD1* and *SLC6A3*, which have previously been found to be associated with schizophrenia. An epigenetic study of monozygotic twins discordant for schizophrenia identified a number of loci differentially methylated in peripheral blood.^12^ Selected gene promoters have also been analysed for differential DNA methylation in the brain tissue from small numbers of patients with schizophrenia. Some of these genes include *RELN*,^13,14^ *COMT*,^15^ *SOX10*^16^ and *HTR2A*.^17^ An earlier study of 12 000 CpG islands in the frontal cortex of 35 schizophrenia and 35 controls revealed differential DNA methylation in genes associated with glutamatergic and GABAergic pathways.^18^ Apart from the present study, the only extant study using a 450 000 genome-wide methylation array was performed in leukocytes from patients with schizophrenia.^19^

DNA methylation analysis of schizophrenia has been more widely performed in peripheral tissue, because it can be readily obtained from living patients. The epigenetic profile differs in the brain compared with the peripheral tissue; however, some regions may have common patterns,^20^ which would make these regions ideal as potential biomarkers for schizophrenia. Some of the genes found to be differentially methylated in peripheral tissue of schizophrenia patients include *HTR1A*,^21^ *HTR2A*,^22^*BDNF*,^23^ *GRM2*,^24^ *GRM5*^24^ and *COMT*.^25,26^

Brain tissue from the Human Brain and Spinal Fluid Resource Centre, CA, USA, was obtained in order to examine tissue involved in the etiology of schizophrenia. We analysed this tissue in a genome-wide methylation study of schizophrenia. We report significant differences in methylation status in brain tissue from schizophrenia patients compared with that from controls. In addition, unsupervised clustering analysis revealed two distinct groups corresponding to schizophrenia and controls. Results of future epigenetic studies hold great promise of a schizophrenia biomarker and treatment, as epigenetic processes can be reversed.

## Materials and methods

### Samples

Frontal cortex post-mortem brain tissue from individuals with Diagnostic and Statistical Manual of Mental Disorders, 4th Edition-diagnosed schizophrenia (*n*=24) and controls (*n*=24) was provided by the Human Brain and Spinal Fluid Resource Centre (courtesy of James Riehl). Each sample consisted of a coronal section (7-mm thick) that had been quick frozen, and a section of frontal cortex was dissected from each frozen section sample weighing (0.4–1.0 g). Demographic data, including age, post-mortem interval (PMI) and gender, are summarised in Table 1. PMI in our study is defined as the time between death and when the brain section is quick frozen. The mean (±s.d.) time between death and the tissue stored at 4 °C was 4.48±3.86. All but two of the schizophrenia subjects were known to be receiving antipsychotic medication at time of death. Cause of death of five schizophrenia patients was suicide. Extraction of DNA was performed at the UCLA Clinical Microarray Core Laboratory (Los Angeles, CA, USA) using the Roche MagNa Pure Compact (Roche, CA, USA). The quality and quantity of DNA was assessed using spectrophotometry, and was found to be satisfactory for all samples. Ethics approval for the project was obtained from the Human Research Ethics Committee of the Queensland University of Technology.

### Illumina Infinium HumanMethylation450 Beadchip

DNA samples were sent to the Australian Genome Research Facility and stored at −20 °C. Quality checking of the samples was performed by Nanodrop Spectrophotometer (Nanodrop, Wilmington, DE, USA) and resolution on a 0.8% agarose gel. Samples were bisulphite converted with Zymo EZ DNA Methylation kit (Zymo Research, Irvine, CA, USA). GenomeStudio v2011.1 (Illumina, San Diego, CA, USA) with Methylation module 1.9.0 software with the default Illumina settings and Illumina HumanMethylation450_15017482_v.1.2 manifest files was used in the methylation analysis. The Infinium platform assays more than 485 000 CpG sites, encompassing 99% of RefSeq genes. It covers 96% of CpG islands with multiple sites in the island, the shores (within 2 kb from CpG islands) and the shelves (>2 kb from CpG islands). It also covers CpG sites outside of CpG islands and DNase hypersensitive sites, as well as incorporating miRNA promoter regions. All the Illumina quality controls were found to be in order, which included sample-independent controls, sample-dependent controls, staining controls, extension controls, target removal controls, hybridization controls, bisulphite conversion I and II controls, specificity controls, non-polymorphic controls and negative controls.

### Data processing

For methylation analysis, IDAT files were loaded into the R (2.15) environment using the Bioconductor minfi package (1.4.0).^27^ The arrays were then background and control normalised using the minfi package. Technical differences between Infinium I and Infinium II probes were removed using Subset-quantile Within-Array Normalisation, developed by Maksimovic *et al.*^28^ and available in the minfi package. The methylation status for each probe was recorded as a *β*-value that ranged between 0 and 1, where values close to 1 represent high levels of methylation and where values close to 0 represent low levels of methylation.

A detection *P*-value was calculated for all probes on all arrays. A *P*-value>0.05 indicates that the data point is not significantly different from background measurements. Probes were removed from analysis if >50% of the samples had a detection *P*-value>0.05 (*n*=39).

Next, probes that are designed for sequences on either the X (*n*=11 232) or Y (*n*=416) chromosome were removed. Finally, probes with single-nucleotide polymorphisms present within 10–50 bp from query site (*n*=59 892), and within <10 bp from query site (*n*=36 535) were removed; with overlap, this totalled *n*=89 678 probes. Because of overlap in some of these conditions, *n*=100 345 probes were filtered leaving *n*=385 167 for analysis.

### Differential methylation detection

In order to assess differences in methylation between groups, the original *n*=385 167 *β*-values were converted to *M*-values via the logit transformation as recommended by Du *et al.*^29^ Differentially methylated probes were detected using the limma package.^30^ The limma procedure uses linear models to assess differential methylation, whereby information is shared across probes.^31^ A major benefit of the limma procedure is that it allows the inclusion of covariates (such as age) or other factors (such as PMI) in the specification of the linear model. As such, we were able to adjust for age and PMI in the detection of differentially methylated probes by including age and autolysis covariates in the specification of the design matrix. Although most studies have found that methylation status is unaffected by PMI, we decided to adjust for PMI as a confounder.^32,33^ Probes were considered to be differentially methylated if the resulting adjusted *P*-value was <0.05. The Benjamini–Hochberg method^34^ was used to adjust the *P*-values and ensure that the false discovery rate was <0.05. The corresponding gene list was derived from the gene annotations associated with the probes.

### Unsupervised clustering

For cluster analysis, the top 3000 most variable probes were selected (based on the s.d. of the *β-*value). A recursively partitioned mixture model (RPMM) was used to cluster the *β-*scores. RPMM is a model-based unsupervised clustering algorithm developed for measurements that lie between 0 and 1. This algorithm was implemented using the *RPMM* Bioconductor package.^35^ The implementation of RPMM was identical to Hinoue *et al.*^36^ who used a fuzzy clustering algorithm for initialisation and level-weighted version of Bayesian Information Criterion as a split criterion.

In order to adjust for age and PMI, a series of linear models were fitted to the *M*-values (logit-transformed *β-*values) using the function lmFit in the limma package. Coefficients for age and PMI, along with an intercept were estimated for each probe. Owing to this model specification, the residuals of the linear model represent the methylation values adjusted for the effect of age and PMI. The residuals were then back-transformed and clustered using the RPMM method implemented for the unadjusted probes.

**Figure 1:**
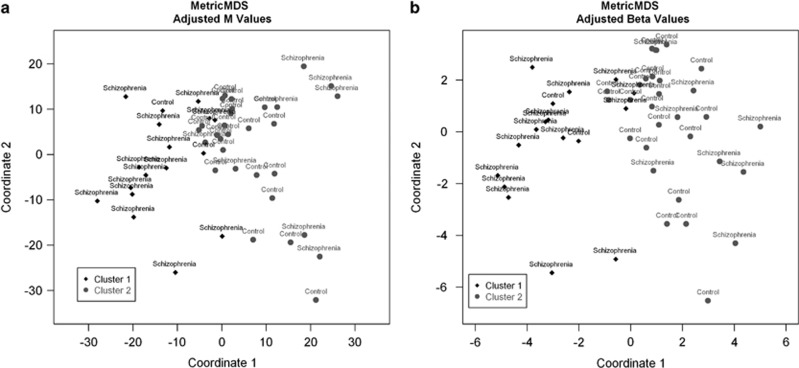
Multidimensional scaling (MDS) plot of the adjusted *M*-values with recursively partitioned mixture model (RPMM) cluster of adjusted *β*-values denoted by black (cluster 1) or grey (cluster 2) (**a**). MDS plot of the adjusted *β*-values with RPMM cluster of adjusted *β*-values denoted by black (cluster 1) or grey (cluster 2) (**b**).

To allow visualisation of the distance between samples and to further reinforce the RPMM clustering, multidimensional scaling with a Euclidian distance metric was performed on both the adjusted *M*-values (before back transformation) and the adjusted *β-*values. The first two coordinates, along with the RPMM clusters, are visualised in Figures 1a and b.

## Results

### Differential methylation

Between the 24 controls and the 24 schizophrenia samples, a total of 19 582 probes were identified as differentially methylated (Supplementary Table 1). Approximately 55.9% of probes were hypomethylated in the schizophrenia group. Of those probes, 2536 were in a promoter-associated region (surrounding a gene transcription start site) and 1443 were both promoter-associated and located at a CpG island (Table 2). However, as the ages and PMI were significantly different between the controls and the schizophrenia patients, differential methylation analysis was performed again, this time adjusting for age and PMI. One sample from a patient with schizophrenia did not have age, sex or PMI recorded, and hence they were excluded from the adjusted analysis.

After adjusting for age and PMI, 4641 probes were declared to be differentially methylated, with ~47.3% hypomethylated in the schizophrenia group. The 4641 probes corresponded to 2929 unique genes (Supplementary Table). Of those genes, 817 were promoter-associated and 599 were both promoter-associated and located at a CpG island. Furthermore, of the 599 genes both promoter-associated and located at a CpG island, 491 were in common with the unadjusted results (Table 2).

**Figure 2:**
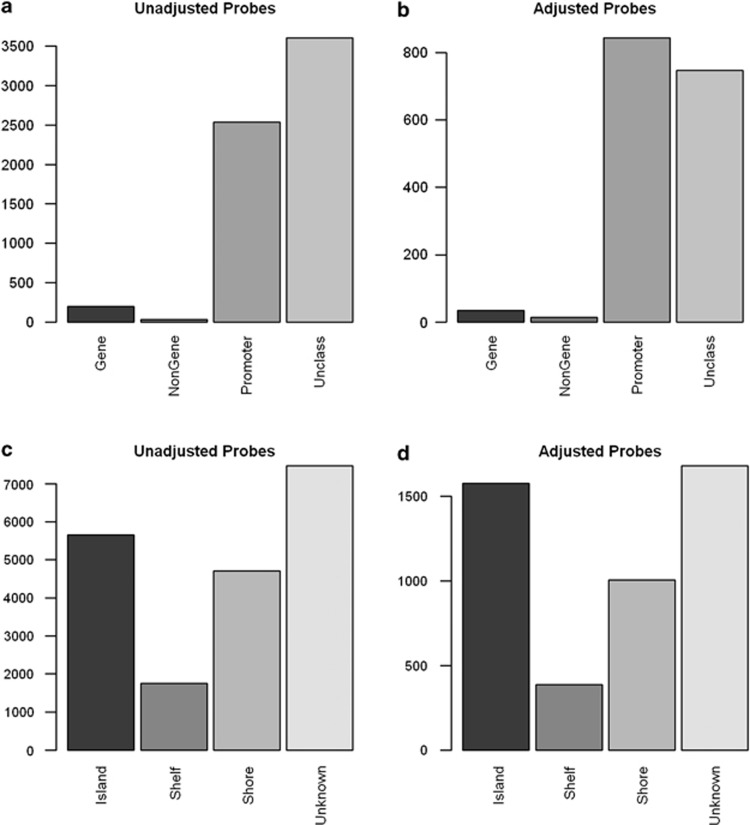
Probe relation to CpG island and probes regulatory feature group. Number of probes found in regions of gene associated, non-gene associated, promoter associated and unclassified, both (**a**) unadjusted and (**b**) adjusted for age and post-mortem interval (PMI) (excluding those with no regulatory feature group information *n*=13214 and *n*=3002, respectively). Number of probes found in CpG islands, shelves (north and south) and shores (north and south), including those with no CpG island information (unknown), both (**c**) unadjusted and (**d**) adjusted for age and PMI.

As expected, altered DNA methylation mostly occurred in the CpG islands (29% unadjusted, 34% adjusted; Figure 2) The proportion of probes in a CpG island found differentially methylated in the adjusted results is significantly higher than the overall proportion of probes found in CpG islands on the array as a whole (overall 30.9% *P*=1.23 × 10^−5^). Furthermore, a high percentage of aberrant DNA methylation occurred in promoter-associated regions, (13.0% unadjusted, 18.2% adjusted; Figure 2). The proportion in the unadjusted results is significantly smaller than the proportion of promoter regions found on the arrays as a whole (overall 20.2% *P*=7.1 × 10^−4^).

The adjusted and unadjusted lists have many genes in common with previously published results. The largest gene list available is that of Nishioka *et al.*^37^ who published a list of 589 unique genes associated with probes significantly differentially methylated between people with first-episode schizophrenia and controls. Of the 589 genes found differentially methylated in the peripheral blood samples, 99 overlap with the adjusted list. Seven of the 589 genes found by Nishioka *et al.*^11^ were found by Reinius *et al.*^38^ to be differentially methylated between leukocyte subtypes, which would also explain differences in the two lists.

**Figure 3:**
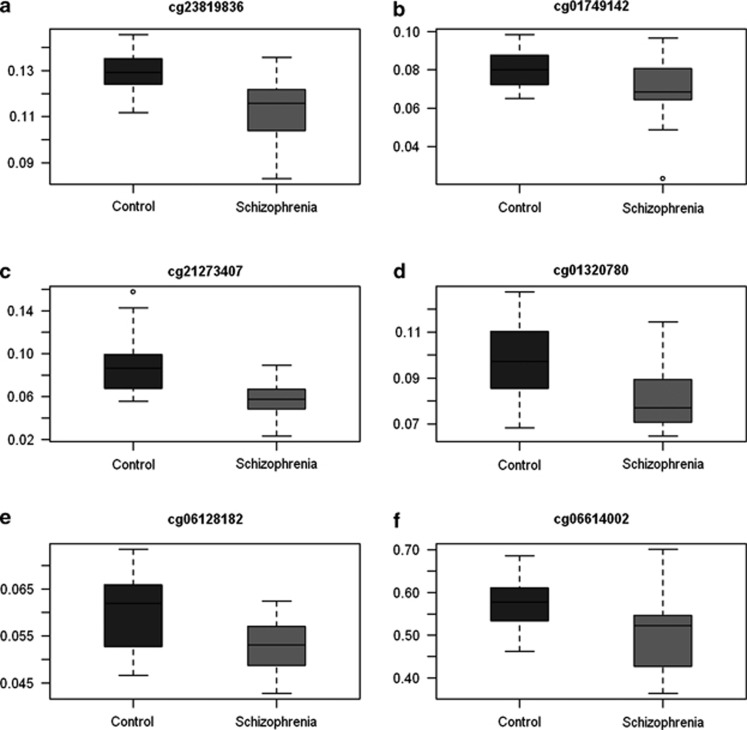
Box plots of *β*-values for the control and schizophrenia groups for probes associated with genes of interest. The median *β*-value is denoted by the solid middle line (**a**) ‘cg27026005' is promoter associated (PA) and located on a CpG island, it is associated with *AKT1.* (**b**) ‘cg01749142' is also PA and located on a shore, it is associated with *AKT1*. (**c**) ‘cg21273407' is located on a CpG island, it is associated with *NOS1*. (**d**) ‘cg23401624' is located on a CpG island and is PA, it is associated to *DNMT1*. (**e**) ‘cg06128182' is located on a CpG island and is PA, it is associated to *DNMT1*. (**f**) ‘cg06614002' is located on a shore and is associated with *SOX10*.

A gene list was generated based on genes previously found to be associated with schizophrenia from genetic and/or DNA methylation studies.^11,12,19,37^ This gene lists includes *DRD2*, *NOS1*, *AKT1*, *HTR2A*, *SOX10*, *FOXP2*, *DTNBP1*, *NRG1*, *PPP3CC*, *BDNF*, *ZNF804A*, *NRGN*, *DRD4*, *MGST1*, *COMTD1* and *GABRB2*. Genes identified as differentially methylated after adjustment include *NOS1*, *AKT1*, *DNMT1*, *SOX10*, *DTNBP1* and *PPP3CC*, whose distributions can be seen in Figure 3.

### Cluster analysis

Clustering of the unadjusted *β*-values revealed three distinct groups, two containing mostly those with schizophrenia (78% and 100%, respectively), whereas the other group contained a mixture of the two sample types (Table 3).

Clustering of the age and autolysis-adjusted *β*-values revealed two distinct groups, one containing mainly schizophrenia patients (88%), the other containing mainly controls (73% Table 3). Cluster 1 had significantly more patients with schizophrenia compared with controls (*χ*^2^=14.1; df=1; *P*=1.74 × 10^−4^). The multidimensional scaling plots on both the adjusted *M-* and *β-*values (Figure 1) further reinforce the distinction between schizophrenia patients and controls. Interpreting the results from these three analyses in conjunction, we can be conservatively confident that at least 12 out of the 24 patients are clearly distinguishable from controls.

### Differential methylation between schizophrenia groups

The results of the clustering indicate that the methylation profiles in those with schizophrenia are a heterogeneous group. There were some profiles that were consistently deemed distinct from the controls, whereas there were others that were not found to be significantly dissimilar. Twelve samples in particular tended to exhibit the former trait. When comparing these two potential subgroups of those with schizophrenia, we can see that the two subgroups exhibit no obvious difference in characteristics (Table 4). Thus, there is potential for methylation arrays to be used to detect differences within these two potential subgroups.

Differential methylation analysis between the two schizophrenia subgroups indicated that there were 73  222 probes that were differentially methylated (Table 5). Of those probes, 6681 were promoter-associated and 2006 were both promoter-associated and located at a CpG island. After adjusting for age and PM1, 56 001 probes were found to be differentially methylated (Supplementary Table 3), 4779 being promoter-associated and 1238 both promoter-associated and located at a CpG island. The abundance of differentially methylated probes suggests significant groupings within the schizophrenia methylation profile. By contrast, a history of completed suicide or the presence of another psychiatric disorder revealed no significant differences in methylation.

## Discussion

Differential DNA methylation in schizophrenia has been reported in several studies to date, although most of these studies involve the use of non-functional tissues such as blood. In this study, we analysed DNA methylation status in brain tissue, the primary tissue of pathology in schizophrenia, employing a genome-wide methylation array with very extensive coverage of the potential methylation sites in the human genome. After adjusting for age and PMI, 4641 probes corresponding to 2929 unique genes were found to be differentially methylated. When we compared the differentially methylated gene list with past studies using peripheral leukocyte samples, we found a high concordance rate, particularly for genes previously found to be associated with schizophrenia. Of the 589 genes Nishioka *et al.*^11^ found to be differentially methylated in peripheral blood cells from patients with schizophrenia, we were able to replicate 99 of these in the brain tissue. This shows promise for the use of non-invasive tissue such as blood or saliva to be used as a future diagnostic indicator of schizophrenia. We are aware of only one other study that used the 450 Illumina array in schizophrenia, although peripheral leukocytes were analysed rather than the brain tissue.^19^ That study identified 10 747 differential DNA methylation sites in medication-free subjects.^19^ One of the genes they identified was *RAI1*, which has altered DNA methylation in the present study as well as an earlier schizophrenia study using the brain tissue.^18^ Other genes found to be differentially methylated in both the leukocyte study and the present brain tissue study includes *HDAC4*, *GFRA2* and *GDNF.* The leukocyte study did not replicate *COMTD1* and *HTR1A* that were found to be differentially validated from a previous study in the peripheral tissue.^11,19^ However, we report that these genes are differentially methylated in the brain. Although we were able to validate many of the previously identified CpG sites, experimental validation using an alternative method, such as pyrosequencing, would also confirm our results. Functional significance of genes found to be differentially methylated should also be tested by gene expression.

Unsupervised clustering of the top 3000 most variable probes revealed two distinct groups after adjusting for age and PMI. Cluster 1 comprised 88% patients with schizophrenia and 12% controls, whereas cluster 2 comprised 27% patients with schizophrenia and 73% controls. To our knowledge, this is the first report of DNA methylation profiling that is able to significantly differentiate between those with schizophrenia and control subjects. Although Nishioka *et al.*^11^ was able to identify site-specific DNA methylation changes in patients with schizophrenia, they were unable to discriminate between controls and schizophrenia patients using unsupervised clustering.^11^ DNA methylation patterns differ in brain cells compared with peripheral tissues such as blood,^20^ and this may explain the lack of separation reported by Nishioka *et al.*^11^ Although some genes may have the same epigenetic profiles in peripheral and brain tissue, a more comprehensive list of tissue-specific genes may be required to differentiate controls from those with schizophrenia. Another reason may be the analysis of fewer CpG sites (only 27 000 compared with 450 000) in the previous study. This is potentially important, as a DNA methylation signature across the whole genome is required to identify the most important differentially methylated probes. The results of our clustering analysis will need to be confirmed in an independent brain tissue cohort.

Clustering analysis also revealed two subgroups within schizophrenia. It is possible that these two subgroups have specific symptomatology that warrants further investigation in a sample set with a comprehensive clinical history. After adjusting for age and PMI, *DTNBP1, COMT* and *DRD2* were found to be differentially methylated between the two schizophrenia subgroups. Interestingly, these are genes that we have previously found to be associated with schizophrenia.^39, 40, 41, 42^

A recent study has found significant DNA methylation changes in the early stages of development and suggest that aberrant DNA methylation during the transition from the fetal to the postnatal period of development could be critical for the pathogenesis of schizophrenia.^43^ Genes that are differentially methylated from fetal to neonatal life stage include *DRD2, NOS1, SOX10* and *DNMT1*, all of which have been previously found to be associated with schizophrenia. We also found *DNMT1*, *NOS1* and *SOX10* to be differentially methylated in brain tissue from patients with schizophrenia. However, after adjusting for age and autolysis, *DRD2* was not differentially methylated.

The main limitation of our study was that patients were not free of antipsychotic medication, and antipsychotic medication has been shown to influence DNA methylation. A recent study reported that antipsychotic haloperidol was uniquely associated with higher global DNA methylation in patients with schizophrenia, but other antipsychotic drugs were not associated with changes in methylation.^25^ A further study observed that antipsychotics may have anti-inflammatory effects.^44^ However, as patients with chronic schizophrenia are almost certain to be treated with antipsychotic medications, future studies may only be able to adjust for antipsychotic medications in their analyses rather than eliminate these samples entirely. Our samples were obtained from a brain bank and had limited medication history collected. Although controls did not have schizophrenia, they were not screened for other psychiatric conditions such as depression. Antipsychotic medication use was not screened for among the control subjects, as these medications are not commonly used other than to treat psychosis. Another limitation is cell-type heterogeneity in the frontal cortex brain tissue used in our analysis. Our study did not account for differences in cell type seen in the frontal cortex, and a previous study has shown that the two major cell types, neurons and glia, have different DNA methylation signatures.^45^ Statistical methods that estimate brain cell types in gene expression studies^46^ and, more recently, in DNA methylation studies^47^ could be used in future brain DNA methylation studies.

Our data indicate that studies of epigenetic changes in schizophrenia hold promise for the future development of diagnostic and prognostic biomarkers for schizophrenia, as well as therapeutic options that target causative epigenetic alterations. The key is to identify aberrant DNA methylation profiles in a functional tissue and determine if the results can be translated back into a diagnostically feasible tissue such as blood or saliva. Identifying when DNA methylation changes occur is also important in understanding the origins of schizophrenia. During the critical period of development between pregnancy and birth, altered DNA methylation occurs.^43^ If gene–environmental factors that affect DNA methylation status can be identified, then the incidence of schizophrenia could possibly be reduced by targeting the environmental triggers. To put together all the pieces of the schizophrenia puzzle, gene–environment interactions, as well as how they influence epigenetics, need to be identified. Over the years, there have been numerous studies on the effects of single-nucleotide polymorphisms on mRNA expression in schizophrenia, but very few showing how single-nucleotide polymorphisms affect gene expression through DNA methylation. Investigating the involvement of single-nucleotide polymorphisms and their interaction with the environment, as well as their influence on epigenetics, will benefit our understanding of the pathophysiology of schizophrenia. The identification of enzymes that are capable of mediating DNA demethylation in mammalian cells as targets for therapeutic intervention is an exciting prospect that may hold the key to reversing this debilitating psychiatric illness.^48^

## Figures and Tables

**Table 1:** Demographic data for control and schizophrenia patients

	*Control (*n*=24)*	*Schizophrenia (*n*=23)*^a^	P*-value*
Age, mean (s.d.)	71.3 (9.8)	51.6 (21.6)	<0.001
PMI, mean (s.d.)^b^	14.1 (3.3)	23.8 (10.4)	<0.001
Sex, male (%)	19 (79)	16 (67)	0.67

a One patient did not have age, sex or PMI recorded.

b PMI (Post Mortem Interval) is the time between death and brain tissue quick frozen.

**Table 2:** Number of probes found to be differentially methylated between control and schizophrenia patients, both unadjusted and adjusted for age and PMI

	*Unadjusted*		*Adjusted*		*Common*^a^
	*Probes*	*Genes*	*Probes*	*Genes*	
All	19582	7832	4641	2929	2632
Promoter associated	2536	2084	844	817	677
CpG islands	5650	3336	1575	1291	1083
Promoter associated and islands	1443	1349	572	599	491

Abbreviation: PMI, post-mortem interval.

a Genes in common with unadjusted results.

**Table 3:** Characteristics of those in the RPMM clusters for both unadjusted and adjusted *β*-values

	*Unadjusted*			*Adjusted*	
Clusters (*n*)	1 (9)	2 (5)	3 (34)	1 (17)	2 (30)
Schizophrenia, *n* (%)	7 (78)	5 (100)	12 (35)	15 (88)	8 (27)
Males, *n* (%)	7 (78)	2 (40)	26 (76)	10 (59)	25 (83)
Death by suicide, *n* (%)	3 (33)	0 (0)	2 (6)	2 (12)	3 (10)
Age, mean (s.d.)	44.8 (19.6)	69.8 (5)	65 (18.2)	59.8 (19.3)	62.7 (19.4)
PMI, mean (s.d.)^a^	21.6 (8.7)	20.6 (8.7)	17.8 (12.3)	19.9 (9.0)	18.3 (9.2)

Abbreviations: PMI, post-mortem interval; RPMM, recursively partitioned mixture model.

a PMI is the time between death and brain tissue quick frozen.

**Table 4:** Characteristics of those identified as being in schizophrenia subgroups based on clustering results

*Subgroups*	*1*	*2*
*n*	12	12^a^
Males, *n* (%)	7 (58)	9 (82)
Death by suicide, *n* (%)	3 (25)	2 (17)
Age, mean (s.d.)	51.4 (20)	51.8 (24.1)
PMI, mean (s.d.)	22 (10.2)	25.7 (10.8)

a One person in this group did not have sex, age or post-mortem interval recorded.

**Table 5:** Number of probes found differentially methylated between schizophrenia subgroups

	*Unadjusted*		*Adjusted*		*Common*^a^
	*Probes*	*Genes*	*Probes*	*Genes*	
All	73 222	14 701	56 001	12 625	12 501
Promoter associated	6681	3979	4779	2934	2851
CpG island	10 628	4450	7598	3218	3106
Promoter associated and island	2006	1546	1238	919	861

a Genes in common with unadjusted results.
